# Associated factors of labor satisfaction and predictor of postnatal satisfaction in the north-east of Peninsular Malaysia

**DOI:** 10.1371/journal.pone.0238310

**Published:** 2020-08-28

**Authors:** Fatin Imtithal Adnan, Norhayati Mohd Noor, Nor Akma Mat Junoh

**Affiliations:** Department of Family Medicine, School of Medical Sciences, Universiti Sains Malaysia, Kota Bharu, Kelantan, Malaysia; Lausanne University Hospital: Centre Hospitalier Universitaire Vaudois (CH), SWITZERLAND

## Abstract

**Introduction:**

Identifying the factors contributing to maternal satisfaction is a proxy measure to improve the quality of care. It evaluates the health service provision by understanding maternal perceptions and expectations and promoting adherence to health services. This study aimed to identify the sociodemographic, obstetric, and medical factors contributing to labor satisfaction among postpartum women and examine the association between labor and postnatal satisfaction.

**Methodology:**

A cross-sectional study using systematic random sampling in a ratio of 1:5 based on the delivery list in a labor room in a tertiary hospital was applied. Information was obtained from medical records for sociodemographic characteristics and obstetric and medical histories. Face-to-face interviews were performed to obtain responses for Malay versions of the Women’s Views of Birth Labour Satisfaction Questionnaire and the Women’s Views of Birth Postnatal Satisfaction Questionnaire. Simple and general linear regression analyses were performed.

**Results:**

A total of 110 participants responded, accounting for a response rate of 100%. High-risk color codes, the period of gestation, household income, and were significantly associated with maternal satisfaction during labor. The association between labor and postnatal satisfaction was significant.

**Conclusion:**

Identifying these associated factors and differences may lead to understanding and contributing to specific and targeted strategies for tackling issues related to maternal satisfaction.

## Introduction

Maternal satisfaction is multifactorial. Many studies have been dedicated to identifying the most determinant factors [1]. Research conducted in various countries, including the United Kingdom, Australia, Canada, and Sweden, have shown that continuous support from caregivers, a close relationship with them, and a warm atmosphere in the maternity centers as factors that encourage women to obtain more information, participate in decision-making and gain greater overall satisfaction [2–4]. Specific scales and predictors aiming at the highest psychometric quality to measure overall maternity care satisfaction have identified additional factors that shape it, such as the presence of a midwife, the place of birth, woman–caregiver empathy, the amount and quality of the information received and feeling in control of the situation [1].

Developing countries account for 99% of maternal mortality annually [5]. A review of maternal satisfaction in these countries revealed that several factors contributed to the determinants of women’s satisfaction with maternity care. It includes structural elements such as pleasant physical environment, adequate human and medicinal resources; process determinants such as interpersonal behavior, perceived provider competency, and emotional support; and outcomes related determinants such as the health of mothers and newborns. Other factors that also influenced perceived maternal satisfaction were socioeconomic status, access, cost, and reproductive history [6]. Maternal satisfaction, in general, is closely related to expectations and personal experiences. Expectations are mainly influenced by the women’s sociodemographic profile, while own experiences include her obstetric and medical history.

Malaysia’s achievement in maternal health has been lauded as a model for other developing countries because of its improvements in maternal health and sustained decline in maternal mortality ratio (MMR) over the years [7]. This has been primarily due to Malaysia’s significant quantum leap in maternal health as a result of the adequate allocation of resources that includes financial, human resources, and physical infrastructure [8]. However, Malaysia has yet to achieve the MMR target of 11 per 100,000 live births of the Millennium Development Goal [9]. Maternal mortality and morbidity reflected an inadequacy in the quality of care of maternal health.

Different instruments were developed to determine women’s satisfaction with labor and childbirth for English-speaking women [10–13]. These questionnaires assess the woman’s satisfaction with childbirth as a multidimensional construct, with each dimension, including various aspects relevant to measuring satisfaction [14]. The Women’s Views of Birth Labour Satisfaction Questionnaire (WOMBLSQ) is the oldest and remains a sound instrument that not only assesses the dimensions which are common to most scales but also pain relief dimension that has been suggested as a critical factor for satisfaction [15]. The (WOMBLSQ) [12] and Women’s Views of Birth Postnatal Satisfaction Questionnaire (WOMBPNSQ) [13] are multidimensional and sensitive to differences in settings and between women. It has the capacity to reflect the most relevant aspect of maternal satisfaction with care during labor. It can be utilized to compare and contrast satisfaction with different models of care or configurations of services or to assess changes over time [16]. However, there is no gold standard instrument for assessing satisfaction with labor or childbirth [15, 17].

Understanding the level of maternal satisfaction and its contributing factors is important for several reasons. First, reporting the level of maternal satisfaction during labor reflects the quality of care during delivery in tertiary centers. Second, identifying the factors contributing to maternal satisfaction is a proxy measure to improve the quality of care. Third, maternal satisfaction influences the mothers’ outcome. High satisfaction with the quality of care during labor is associated with increased adherence to and better continuity of care helps women in the planning of maternity care and facilitates positive adjustment [18].

This study aimed (i) to identify the sociodemographic, obstetric, and medical factors contributing to labor satisfaction and (ii) to determine whether labor satisfaction is a predictor of postnatal satisfaction. Here, labor satisfaction refers to the satisfaction reported soon after giving birth, that is, prior to discharge from the hospital, and postnatal satisfaction refers to the satisfaction reported one month postpartum. We hypothesized that sociodemographic, obstetric, and medical factors significantly influence labor satisfaction. In this study, satisfaction is defined as a value-based judgment and results from the comparison between expected and perceived services [19].

## Materials and methods

### Population and sample

This study was conducted in Kelantan, which is a state in the northeast of Peninsular Malaysia, with approximately 1.7 million populations. Approximately 32% of the population lives in Kota Bharu, the capital city of Kelantan. There is one state, one teaching, eight district, and three private hospitals providing obstetric services in Kelantan. In 2016, there were 24,170 live births in Kelantan, and the maternal mortality ratio was 29.7 per 100,000 live births. Once after childbirth and the mother has made her initial recovery, she goes home and is visited by the community nurse. She typically sees her healthcare provider in the health clinic at the 4-week postpartum check-up.

**Fig 1:**
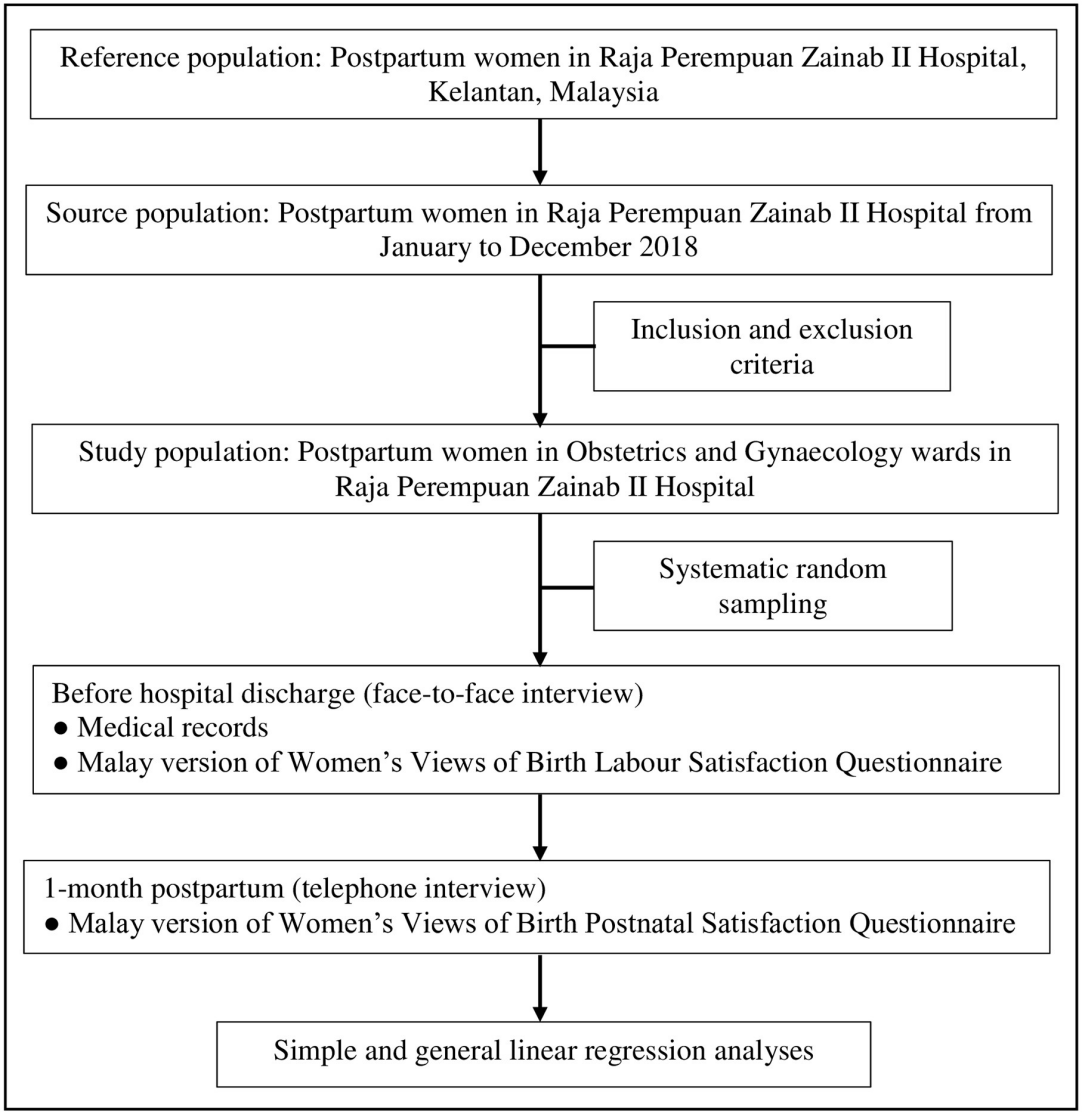
Flow chart of study.

This cross-sectional study included postpartum women in the only one state tertiary referral hospital in Kelantan, Malaysia. Women with high-risk pregnancies as well as low-risk pregnancies but developed complications deliver in this hospital, which accounted for approximately 40% of total deliveries in Kelantan. The reference population consisted of postpartum women in the hospital. The source population included women who were admitted to the hospital from January to December 2018, were aged 18 or older and were able to understand and speak the Malay language. Women who delivered at other birth centers, had a history of diagnosed psychiatric disorder, and were non-Malaysian citizens were excluded. Women with a history of psychiatric disorders were excluded on the grounds that they are vulnerable, their condition cannot be determined to be competent and inability to comply with follow-up. The exposure and the outcome of interest were determined concurrently [20]. Following childbirth, labor satisfaction was assessed, and its possible associated factors were identified. A repeat cross-sectional study was performed one month postpartum to assess postnatal satisfaction (Fig 1).

Sample size calculation to identify the sociodemographic and obstetric factors of labor satisfaction following childbirth was performed by comparing two means for categorical variables and by linear regression for numerical variables using the PS software. The standard deviation (SD) of the satisfaction score among women delivering by cesarean section was 16.89 [21]. An expected detectable difference in a population mean of 10 was decided after considering its clinical importance. The ratio of vaginal to cesarean delivery was one. Taking an alpha of 0.05 and a power of 80%, the minimum required sample size was 92. After assuming a non-response rate of 20%, the calculated sample size required was 110 postpartum women. On average, the number of deliveries in Raja Perempuan Zainab II Hospital is 50 per day. Systematic random sampling in a ratio of 1:5 was used based on the delivery list in the labor room.

### Research tools

Information was obtained from medical records and face-to-face interviews. The case report form contained responses on (i) sociodemographic characteristics, (ii) obstetric and medical histories, (iii) a Malay version of the WOMBLSQ, and (iv) a Malay version of the WOMBPNSQ. The sociodemographic characteristics obtained included information on age, ethnicity, education level, employment status, and household income. The obstetric and medical histories included information on parity, the status of pregnancy, antenatal booking, the period of gestation, bad obstetric history, maternal morbidity status, color code, type of delivery, fetal outcome, the weight of baby, and medical comorbidity.

Period of gestation refers to the duration of pregnancy before childbirth. Bad obstetric history refers to a pregnant mother where her present obstetric outcome is likely to be affected adversely by the previous adverse obstetric events such as repeated abortions, repeated intrauterine deaths, premature delivery, neonatal death and Rh incompatibility.

Severe maternal morbidity refers to potentially life-threatening conditions during pregnancy, childbirth, or after the termination of pregnancy from which maternal near-miss cases would emerge [22, 23]. Maternal morbidity status refers to the presence of at least one of the severe maternal morbidity conditions, namely, hemorrhagic, hypertensive, other systemic disorders (endometritis, pulmonary edema, respiratory failure, seizures, sepsis, shock, thrombocytopenia, and thyroid crisis) and severe management (blood transfusion, central venous access, hysterectomy, intensive care unit admission, a prolonged hospital stay of more than seven postpartum days, intubation not related to the anaesthetic procedure, return to operating room and laparotomy excluding cesarean section) indicators [24, 25].

Color code is a risk approach strategy for maternal health used in Malaysia since 1989 [26]. It grades antenatal mothers according to the severity of risk during each antenatal care visit using four color codes, namely, white, green, yellow, and red. The white code indicates no or low risk. The green code indicates that the woman needs to be monitored by a senior nurse. Yellow code indicates that the risk needs to be monitored by a doctor. The red code signifies immediate hospital referral and admission. Comorbidity refers to the presence of pre-existing medical conditions, such as hypertension, diabetes, asthma, heart diseases, and thyroid disorders.

#### Women’s Views of Birth Labour Satisfaction Questionnaire

The WOMBLSQ measures maternal satisfaction with care following childbirth. It includes 32 items with 11 dimensions of the childbirth process: professional support (five items), expectations (four items), home assessment (three items), holding the baby (three items), support from spouse/partner (three items), pain during labor (three items), pain after delivery (three items), continuity of care (two items), environment (two items), control (two items), and general satisfaction (two items). The original English version has demonstrated adequate reliability both on a global scale, with a Cronbach’s alpha of 0.89, and on the subscales, with Cronbach’s alpha ranging between 0.62 and 0.91 [12].

The items are rated on a seven-point Likert scale from *totally disagree* to *totally agree*. Items are deliberately very positive or negatively worded to enable the respondents to express minimal dissatisfaction. Dimension scores are generated to allow easy comparisons between individual dimensions, and the total score is generated by adding the scores of all the items. The scores are added with the negatively worded items reversed and converted to percentages so that the minimum possible score is 0%, and the maximum possible score is 100%. Higher scores indicate greater satisfaction [12].

The English version of WOMBLSQ was translated into Malay according to the recommended 10 steps of the Principles of Good Practice for the Translation and Cultural Adaptation Process for Patient-Reported Outcomes, which include forward and backward translation, harmonization, and cognitive debriefing in small groups of five to eight respondents [27]. For the Malay version, the home assessment dimension was removed because it was not related to the local setting, and the general satisfaction dimension was analyzed separately from other satisfaction dimensions. It reported high item reliability and item separation at 0.98 and 7.65, respectively, and good person reliability and person separation at 0.78 and 1.90, respectively. The item difficulty measures from +1.55 to −1.64 logit with a spread of 3.19 logit. Person ability ranges from +4.36 to −0.59 logit with a spread of 4.95 logit [28].

#### Women’s Views of Birth Postnatal Satisfaction Questionnaire

The WOMBPNSQ measures maternal satisfaction with care postpartum. It includes 36 items with 13 dimensions, including three items related to general satisfaction. The 12 specific dimensions are supported from professionals (three items) and partners (three items), social support (three items), care from general practitioners (two items) and health visitors (two items), advice on contraception (three items), feeding the baby (four items), the mother’s health (three items), continuity of care (two items), duration of inpatient stay (three items), home visiting (three items), and pain after birth (two items). Each question is loaded highly onto only one dimension. The original English version has demonstrated adequate reliability on a global scale, with a Cronbach’s alpha of 0.84, and the individual dimensions generally have acceptable or good internal reliability, with Cronbach’s alpha ranging between 0.62 and 0.90. The items are rated on a seven-point Likert scale from *totally disagree* to *totally agree*. The total score is converted to a percentage score. Higher scores indicate greater satisfaction [13].

The English version was also translated into Malay according to the Principles of Good Practice for the Translation and Cultural Adaptation Process for Patient-Reported Outcomes [27]. For the Malay version, the social support dimension was removed because its items were not suitable for the local population, and the general satisfaction dimension was analyzed separately from other satisfaction dimensions. It reported item reliability and item separation at 0.99 and 9.02, respectively, and person reliability and person separation at 0.48 and 0.90, respectively. The item difficulty measures from +0.54 to −0.67 logit with a spread of 1.21 logit. Person ability ranges from +0.70 to −0.31 logit with a spread of 1.01 logit [29].

### Data collection

The prospective participants were identified during hospitalization based on the study’s eligibility criteria. They were then briefed and invited to participate in the study. Once they voluntarily agreed to participate, they signed informed consent forms. Participants were recruited until the required sample size was reached. The interviews were done twice; first was face-to-face interview before hospital discharge, and the second was telephone interview at one month postpartum. These interviews were performed by our researcher (FIA). During hospitalization, hospital and home-based medical records were reviewed to retrieve mothers’ information, including contact numbers, and obstetric and medical information in order to determine possible contributory factors of labor satisfaction. Next, a face-to-face interview based on the WOMBLSQ was conducted to obtain information about labor satisfaction. All women preferred to be interviewed at their beds before hospital discharge. The researcher draped the bed with curtains to provide some privacy. The interview method was selected because it is the least burdensome to respondents, requiring only basic verbal and listening skills and no reading skills, as well as the ability to request clarification of ambiguous questions [30]. Then, the women were thanked for their contribution, and bath towels were given as a small token of appreciation. At 1 month postpartum, a telephone interview based on the WOMBPNSQ was conducted to obtain information on postnatal satisfaction. Our researcher (FIA), a medical officer, who was not part of the managing team, obtained consent from the women, extracted information from the medical records, and interviewed them.

### Data entry and analysis

The data were analyzed using SPSS Statistics version 24.0. For Objective 1, simple and general linear regression exploratory analyses were used to identify the sociodemographic and obstetric factors of labor satisfaction following childbirth. The dependent variable was the labor satisfaction score. For Objective 2, simple and multiple linear regression confirmatory analyses were performed. The dependent variable was the postnatal satisfaction score. The independent variable was the labor satisfaction score, and the potential confounders were fetal outcome and parity.

Simple linear regression was done on all independent variables at univariable level. The analysis was continued with multiple linear regression. Variable selection for exploratory analyses was done by automatic backward and forward stepwise procedure. Interaction and multicollinearity were checked in fine modelling. All possible 2-way-interaction terms of the independent variables were done. The model assessment was done by checking the linearity assumptions, equal variance assumption, normality assumption, and outliers by using standardized residual plots. Variables with *P* < 0.05 were considered as statistically significant.

### Ethical consideration

The study was approved by the Human Research Ethics Committee of Universiti Sains Malaysia (USM/JEPeM/17080374) and the Medical Research Ethics Committee of the Malaysian Ministry of Health (NMRR-17-2158-37600). All participants gave written informed consent for the research and that their anonymity was preserved. The women were identified by identification numbers to maintain the confidentiality of the findings.

## Results

A total of 110 participants responded following childbirth, accounting for a response rate of 100%, while 105 participants responded one month postpartum, accounting for a response rate of 95.5%, as five participants could not be reached. Table 1 shows the sociodemographic, obstetric, and medical characteristics of postpartum women. All women were married and lived with their spouses. None had severe maternal morbidity conditions. Seventy women were multiparous; among them, four (4.0%) had complications, and five (4.6%) had experienced fetal death during previous pregnancies.

**Table 1:** Sociodemographic, obstetric, and medical characteristics of postpartum women (n = 110).

Variables	Mean	(SD^a^)	n	(%)
**Sociodemographic**				
Age (years)	29.9	(5.62)		
Household income (RM)	3405.5	(2207.89)		
Ethnicity				
Malay			108	(98.2)
Non-Malay			2	(1.8)
Education level				
Primary and secondary			53	(48.2)
Tertiary			57	(51.8)
Employment status				
Employed			35	(31.8)
Non-employed			75	(68.2)
Partner employment status				
Employed			55	(50.0)
Non-employed			55	(50.0)
**Obstetric and medical**				
Parity	2.6	(1.69)		
Period of gestation (weeks)	38.8	(1.39)		
Hospital stay (days)	2.5	(1.11)		
Weight of baby (kg)	3.0	(0.79)		
Pregnancy status				
Wanted			105	(95.5)
Unwanted			5	(4.6)
Antenatal care booking				
Early (≤12 weeks)			95	(86.4)
Late (>12 weeks)			15	(13.6)
Color code				
White			4	(3.6)
Green			95	(86.4)
Yellow			11	(10.0)
Mode of delivery				
Vaginal			75	(68.2)
Caesarean			35	(31.8)
Fetal outcome				
Alive			109	(99.1)
Dead			1	(0.9)
Comorbidity				
Absent			95	(86.4)
Present			15	(13.6)

^a^ Standard deviation.

### Associated factors of labor satisfaction

The labor satisfaction scores were normally distributed, ranging from 52.5 to 92.0, with a mean (SD) of 73.6 (10.18). In total, 16 variables were chosen for simple linear regression analysis (Table 2) based on their statistical and clinical importance. General linear regression analysis showed that a green color code (*P* = 0.004), a yellow color code (*P* = 0.031), the period of gestation (*P* = 0.039), household income (*P* = 0.001), and comorbidity (*P* = 0.023) were significantly associated with maternal satisfaction during labor. There was no significant interaction between the variables (*P* > 0.05). There was no multicollinearity, as indicated by a variance inflation factor of less than 10. Residual plots for overall model linearity and equal variance assumption, normality assumption, and variable functional form for household income were satisfied.

**Table 2:** Associated factors for labor satisfaction.

Variables	SLR^a^			GLR^b^		
	b^c^ (95% CI^d^)	*t* stat^e^	*P* value	Adj. b^f^ (95% CI^d^)	*t* stat^e^	*P* value
**Sociodemographic**						
Age (years)	−0.008 (−0.35, 0.34)	−0.04	0.965			
Household income (RM)	−0.001 (−0.00, −0.00)	−3.06	0.003	−0.001 (−0.00, −0.00)	−3.37	0.001
Race						
Malay						
Non-Malay	−10.2 (−24.54, 4.14)	−1.41	0.161			
Education level						
Primary and secondary						
Tertiary	2.9 (−0.98, 6.69)	1.48	0.142			
Employment status						
Employed						
Non-employed	2.7 (−1.38, 6.86)	1.32	0.190			
Partner employment status						
Employed						
Non-employed	−0.2 (−4.08, 3.65)	−0.11	0.913			
**Obstetric and medical**						
Parity	0.8 (−0.38, 1.90)	1.32	0.190			
Period of gestation (weeks)	1.6 (0.25, 2.97)	2.35	0.021	1.3 (0.07, 2.60)	2.09	0.039
Hospital stay (days)	−0.2 (−1.90, 1.59)	−0.18	0.860			
Weight of baby (kg)	-0.4 (-2.63, 1.78)	-0.38	0.705			
Status of pregnancy						
Wanted						
Unwanted	6.8 (−2.35, 16.03)	1.47	0.143			
Antenatal care booking						
Early (≤12 weeks)						
Late (>12 weeks)	1.1(−4.55, 6.71)	0.38	0.705			
Color code						
White						
Green	14.4 (4.45, 24.48)	2.86	0.005	13.8 (4.46, 23.10)	2.93	0.004
Yellow	12.3 (0.84, 23.74)	2.13	0.036	11.8 (1.07, 22.50)	2.18	0.031
Mode of delivery						
Vaginal						
Caesarean	−0.9 (−5.05, 3.24)	−0.43	0.667			
Fetal outcome						
Alive						
Dead	4.2 (−16.14, 24.58)	0.41	0.682			
Comorbidity						
Absent						
Present	−6.4 (−11.91, −0.90)	−2.31	0.023	−5.9 (−11.06, −0.82)	−2.30	0.023

^a^ Simple linear regression.

^b^ General linear regression.

^c^ Crude regression coefficient.

^d^ Confidence interval.

^e^ *t* statistic.

^f^ Adjusted regression coefficient.

### Association between labor and postnatal satisfaction

The postnatal satisfaction scores were normally distributed, ranging from 41.4 to 81.3, with a mean (SD) of 59.5 (9.17). The association between labor and postnatal satisfaction was significant (*P* = 0.029). It was also significant (*P* = 0.045) after adjusting for fetal outcome and parity (Table 3). There was no significant interaction between the variables (P > 0.05). There was no multicollinearity, as indicated by a variance inflation factor of less than 10. Residual plots indicated that overall model linearity and equal variance assumption, normality assumption, and variable functional form for parity were satisfied.

**Table 3:** Association between labor and postnatal satisfaction.

Variable	SLR^a^			MLR^b^		
	b^c^ (95% CI^d^)	*t* stat^e^	*P* value	Adj. b^f^ (95% CI^d^)	*t* stat^e^	*P* value
Labor satisfaction	0.19 (0.021, 0.368)	2.22	0.029	0.18 (0.003, 0.359)	2.03	0.045

^a^ Simple linear regression.

^b^ Multiple linear regression confirmatory analysis after adjusting for fetal outcome and parity.

^c^ Crude regression coefficient.

^d^ Confidence interval.

^e^ *t* statistic.

^f^ Adjusted regression coefficient.

## Discussion

This study explored several sociodemographic, obstetric, and medical factors of labor satisfaction. Green and yellow color codes, long gestation period, low household income, and absence of comorbidity were associated with a high level of labor satisfaction among postpartum women.

Several researchers have developed many high-risk rating systems aimed at reducing perinatal mortality in pregnant women over the past 40 years. [31–37]. Such rating systems are mainly numerical. The overall risk score is calculated to obtain a composite score and then categorize women into two or more categories, from low to moderate and high-risk groups [31, 38, 39]. The issue with this summing up is that different factors carry different risks. It is very unlikely that the risk increases as a simple arithmetic progression according to the number of factors present [39].

The WHO has developed a classification method (a screening tool) to classify high-risk women from the basic component of the antenatal care program [40]. A 1992 review of the WHO Maternal Health and Safe Motherhood Research program concluded that risk screening has failed to fulfil its goals of effective antenatal risk screening to prevent adverse maternal outcomes [41]. According to the WHO, risk evaluation methods widely used in the 1990s relied mainly on sociodemographic and physical features to identify women and did not necessarily indicate obstetric complications [40]. Risk screening program had a likelihood ratio of 1.16, suggesting that it was unsuccessful in identifying women at risk of pregnancy complications and generating a risk group too large for referral [42]. Twelve rating tools were found to be poorly performing and resulted in more frequent hospitalizations and community interventions labelled ‘at risk’ with no substantial improvement in premature birth rates [43]. Many of the high-risk risk factors only showed a relationship with adverse effects with no evidence of actual causation [44].

The risk stratification approach using color codes were used in Malaysia since 1989 [45]. Color coding is a method of translating information into color to convey it faster. Color coding in antenatal care was adopted in Malaysia in 1989 [46]. The risk-oriented antenatal care strategy is applied in such a way that women are monitored using color codes in order to rapidly identify the degree of risk of complications and take immediate appropriate action during a specific gestational period [46–48]. The four colors that have been used to classify the risk level are white (no risk), green (low risk), yellow (high risk), and red (extremely high risk) [26]. Based on the risk stratification, a greater number of appointments and health care visits is correlated with a higher risk, which warrants more attention by the health workers. Those at a higher risk, especially with a red color code, may need to be referred to a secondary or tertiary center. In this study, green and yellow codes were associated with greater satisfaction compared to white. In the Malaysian antenatal health system, women with a white tag are followed up by nursing staff, while those with green and yellow tags should be seen by medical officers and family medicine specialists, respectively, in clinics [48]. A red code necessitates immediate referral to the hospital [48]. In other words, women with codes other than white are given more attention by health workers, which can lead to higher levels of labor satisfaction. On the contrary, those with a red code might have higher levels of stress. However, this cannot be confirmed by our findings, as there were no red-coded women identified.

A healthy child is the dream of every mother. Children born at term are associated with better prognostic outcomes compared to children born preterm. Women with poor childbirth outcomes have higher stress levels, which in turn, lead to trauma [49, 50] and poor labor satisfaction [51, 52]. Longer gestation (infants born full-term within 37–41 weeks gestational age) has tremendous benefits both for women and infants because it is associated with reduced morbidity and mortality of infants [53]. Puerperium is associated with changes in physiological and psychological functions and may affect maternal health even when these processes are normal. Physiological changes may resolve shortly after baby delivery, while other changes may detract from postpartum recovery. The arrival of a preterm baby is often extremely stressful and may become a traumatic time for parents [54].

In Malaysia, access to public medical services, including maternity care, is heavily subsidized and citizens do not have to pay much for the ward and medical treatment. Women can opt for some comfort and convenience of a private hospital that cost between RM 2,000 and RM 5,000 depending on the mode of delivery. However, in Kelantan, 85% of households earn below RM 7,000 that is lower than the monthly national average household income of RM 6,958 [55]. Income, expectations, and perceptions are interconnected [6]. Expectations and perceptions affect satisfaction, which is a mirror image of expectation fulfillment.

In this study, we found that for every RM 1000 increase in household income, there is a 1.3 percentage unit decrease in the satisfaction score during labor. Women with lower income being negatively correlated with satisfaction are consistent with the literature [56–59]. A similar study showed that lower income is associated with higher labor satisfaction [56]. Mothers from lower socioeconomic status generally had a poor expectation and were easily satisfied. They would assess their labor as ‘very good’[57] and had a higher level of satisfaction with the hospital environment [60].

Whereas, women with high income are likely to have better perception and, therefore, higher expectations. As high expectations are hard to fulfill, some women might get disappointed [6]. One study found that the higher the income, the more satisfied the woman was [61]. The higher satisfaction is particularly related to the health care providers’ attitude [60], and these facts imply that the service provided to the woman during labor has to be more patient-centered. Some studies found no association between household income and maternal satisfaction [51, 62–64]. Satisfaction is multidimensional [3]; a single factor may not weigh on it, and therefore, it should be examined from a broad perspective.

Non-communicable diseases have become a heavy burden for the healthcare system. In Malaysia, the second most common cause of death among women in the general population is ischemic heart disease, which accounted for 10.5% in 2017—second only to pneumonia [65]. In general, women with underlying comorbidity are subject to certain limitations that affect their satisfaction. Along with pregnancy, medical comorbidity can give rise to various unpredictable complications [66, 67]. These can cause tension, anxiety, and frustration [68], which directly influence labor satisfaction. In this study, women with underlying medical conditions reported having decreased levels of labor satisfaction. This finding is consistent with other studies reporting that comorbidity had a negative influence on the level of labor satisfaction [63, 69]. One study found that comorbidities like HIV, hypertension, and diabetes mellitus are associated with a 3.1% decrease in labor satisfaction [69]. The absence of comorbidity has a positive influence on maternal satisfaction [70]. This information will help in planning and implementing patient-centered care during labor and postnatal to improve maternal satisfaction.

This study found that age did not affect labor satisfaction. This finding is in agreement with most of the literature [52, 62, 63, 71, 72]. On the contrary, few studies have found that older women have higher satisfaction scores compared to younger women [61, 73]. In contrast, one study reported that younger women were more satisfied [56].

In this study, we did not observe a significant association between education level or employment status with labor satisfaction. Similar findings were also reported among the postpartum Spanish women using the WOMBLSQ [74]. However, many studies have shown that more educated women have poorer satisfaction compared to less educated women [21, 52, 62, 72, 73, 75, 76], and one study reported contradictory findings [61]. Similar to education status, those who are employed also have higher expectations. Several studies have shown that unemployed mothers are more satisfied than employed ones [21, 52, 61, 77]. Labor satisfaction increases when the service provided meets expectations, and the education level indeed increases the awareness of the service and, thus, expectations [56, 75]. However, such expectations were not observed in this study. Local research with a majority (93.3%) of the women had secondary and tertiary education reported satisfaction with the provision of maternity care. They were confident and satisfied with the diagnosis provided by the healthcare providers and had good opinions regarding provider-patient communication and relationships [78].

Multiparas are expected to have higher labor satisfaction compared to primiparas. Women with previous experience of childbirth have more realistic and practical expectations, which are translated into easier labor satisfaction [52, 61, 73, 76, 79, 80]. Primiparas, on the other hand, have more fear and higher expectations [61], thus needing more support and assistance [51], which leads to low levels of satisfaction [51, 62, 71, 72]. This study found no association between parity and labor satisfaction. This suggests that the care provided may have fulfilled the needs and expectations of primiparas and multiparas equally well [2]. Other factors, such as social support during labor, also play a role in influencing labor satisfaction [3, 81].

The status of pregnancy reflects pregnancy intention. Unintended pregnancy is associated with lower life satisfaction and affects maternal mental health [82, 83]. Women who want their pregnancy is more likely to be satisfied [21, 56, 63]. However, this outcome of unintended pregnancy may be confounded by other elements such as socioeconomic status [84]. In this study, there was no significant association between the status of pregnancy and labor satisfaction. This could be because the majority of participants fall into the wanted pregnancy category.

The World Health Organization (WHO) recommends that all pregnant women have their first antenatal care in the first trimester [85]. In the Malaysian health system, early booking for first antenatal care is defined as before 13 weeks of gestation [26]. Globally, it is estimated that in 2013, more than 40% of pregnant women did not receive early antenatal care [86]. Despite the WHO recommendation, studies in a few parts of Malaysia showed a high prevalence of late bookings, especially in rural areas and in western Malaysia [87–89]. In this study, 86.4% of the women had early antenatal bookings. Studies on the association between antenatal booking and labor satisfaction are scarce. This study’s findings suggest a non-significant relation between antenatal booking and labor satisfaction. Nonetheless, studies on the relationship between antenatal care follow-up and labor satisfaction have reported positive correlations [21, 56, 75]. Health knowledge during pregnancy is very important, as it is one of the factors that influence the acceptance and utilization of health facilities. Other than sound knowledge regarding pregnancy and its complications, planning throughout pregnancy empowers the women and therefore increases satisfaction [88].

Many studies have reported a relationship between the mode of delivery and labor satisfaction. However, in this study, no association was found. It is not uncommon that complications in pregnancy at the time of delivery are dealt with cesarean section. In one local study, two-third of severely morbid women who had cesarean section reported acceptance towards difficulties faced during childbirth [78]. It is because of adaptation to physical and emotional experiences through religious reasoning and maternal disposition. The adaptation and acceptance of circumstances may help to explain the non-significant finding between mode of delivery and satisfaction. One study found that vaginal delivery offered more satisfaction based on WOMBLSQ than instrumental or cesarean delivery [12]. Similar findings have been reported by other studies [69, 72, 73, 76]. In contrast, one study found that delivery via cesarean section offered higher labor satisfaction [77]. Comparing instrumental and non- instrumental vaginal delivery, studies showed that instrumental delivery led to poorer labor satisfaction [63, 69].

This study was conducted in a referral hospital that received both high and low-risk pregnant mothers. Among the 105 women involved during the study period, there was no case of a woman with severe maternal morbidity conditions detected. One local study reported that only 1.8% of the 21,756 population studied had severe maternal morbidity [90]. In this study, we found no association between fetal outcome and labor satisfaction. This finding may not reflect the actual relation, given that the proportion of participants with good fetal outcomes is 99.1%. One study reported that fetal outcome was positively associated with maternal satisfaction, even with a high proportion of alive fetuses (88.7%) in a sample of 368 participants [56].

Between labor and postnatal visit at 4 weeks postpartum, continuity of care is maintained by the primary health care providers at the district level. In this study, we found an association between labor and postnatal satisfaction. Satisfaction at childbirth fosters a good perception and a positive attitude [80], which affect the participants’ assessment of postnatal service. Perception is influenced by previous experiences. A good experience during hospitalization can contribute to building trust and confidence toward health services in general. This study showed that the level of postnatal satisfaction could be predicted by the participants’ perceived satisfaction during childbirth. Moreover, an evaluation of labor satisfaction may be more suitable after a certain period of time has passed after childbirth, allowing for some time to reflect. Gathering the mother’s experiences while in the hospital could be problematic because they may be physically and psychologically vulnerable [15].

Satisfaction is a meaningful output indicator for the quality of care. Assessment of the satisfaction with maternity services is crucial, particularly satisfaction with care during labor [91, 92]. Labor satisfaction is important for health providers in providing continuous high-quality care in maternal health [57, 93]. Providing high quality of care in maternity services involves giving women the best possible medical care and outcome during antenatal, labor, and postnatal period [94]. Determinants of maternal satisfaction covered all dimensions of care across structure, process, and outcome [95].

The collecting, understanding, and acting on information received in regards to maternal satisfaction is the basis for improvement in maternity services. To implement evidence-based practice in labor and postnatal care, satisfaction measurement tools, and their timing are important. The assessment needs to be done using a valid and reliable questionnaire that matches the setting and population tested [96]. Satisfaction with maternity care, too, can change over time. Different ratings could be produced even over a short period whilst the mother is still in hospital compared to after discharge [91].

Among the strengths of this study are; it applied established and clear definitions and standard identification criteria of severe maternal morbidity. The maternal morbidity has been considered as an alternative indicator for the evaluation and improvement of maternal healthcare services other than maternal mortality [24]. This research uses questionnaires that can reflect the most relevant aspects of maternal satisfaction with care during labor and the postnatal period, such as provision and continuity of care, women’s expectations, partner support, and pain management, and has been tested with various models of maternity care. These questionnaires were translated into Malay; their psychometric properties were assessed and were the first maternal satisfaction questionnaires to be used in the Malaysian population. This study has some limitations. The assessment of satisfaction before discharge from the hospital reported a higher level of satisfaction, and this labor satisfaction scale was not used again during the telephone interview to be able to compare levels. The follow-up interview was conducted through telephone, during which participants may not be able to focus on a lengthy questionnaire, which may also affect the results. There is no gold standard for measuring birth or labor satisfaction that can be used for comparison or validation of the questionnaires used. The dimensions involve in satisfaction, too, may vary between countries and calls for cultural adaptations [15]. Despite these limitations, the questionnaires have been validated on the relevant population and proven to be sufficient for all the analyses performed [28, 29].

## Conclusion

Women’s experiences of labor and postpartum are critical components of the evaluation of maternity healthcare. Labor satisfaction may significantly predict postpartum satisfaction. For clinicians, recognizing medical determinants such as poor obstetrics histories, a shorter period of gestation, and the presence of comorbidity may play an essential role in the design of maternity services and in improving quality. Women with high household income are likely to have higher expectations of care. For researchers, replication of studies in other populations is needed to provide comprehensive cross-cultural exploration to explain the differences in maternal satisfaction caused by different living conditions.

## List of abbreviations

adj. Reg. Coef: Adjusted regression coefficient
CI: confidence interval
SD: standard deviation
WHO: World Health Organization
WOMBLSQ: Women’s Views of Birth Labour Satisfaction Questionnaire
WOMBPNSQ: Women’s Views of Birth Postnatal Satisfaction Questionnaire
